# Mechanism of trifluorothymidine potentiation of oxaliplatin-induced cytotoxicity to colorectal cancer cells

**DOI:** 10.1038/sj.bjc.6603549

**Published:** 2007-01-23

**Authors:** O H Temmink, E K Hoebe, K van der Born, S P Ackland, M Fukushima, G J Peters

**Affiliations:** 1 Department of Medical Oncology, VU University Medical Center, Amsterdam, The Netherlands; 2 Department of Medical Oncology, Newcastle Mater Misericordiae Hospital, Waratah, NSW, Australia; 3 Tokushima Research Center, Taiho Pharmaceutical Co., Tokushima, Japan

**Keywords:** apoptosis, colorectal cancer, combination studies, DNA-strand breaks, oxaliplatin, Pt-DNA adducts, trifluorothymidine

## Abstract

Oxaliplatin (OHP) is an anticancer agent that acts by formation of Platinum-DNA (Pt-DNA) adducts resulting in DNA-strand breaks and is used for the treatment of colorectal cancer. The pyrimidine analog trifluorothymidine (TFT) forms together with a thymidine phosphorylase inhibitor (TPI) the anticancer drug formulation TAS-102, in which TPI enhances the bioavailability of TFT *in vivo*. In this *in vitro* study the combined cytotoxic effects of OHP with TFT were investigated in human colorectal cancer cells as a model for TAS-102 combinations. In a panel of five colon cancer cell lines (WiDr, H630, Colo320, SNU-C4 and SW1116) we evaluated the OHP-TFT drug combinations using the multiple drug–effect analysis with CalcuSyn software, in which the combination index (CI) indicates synergism (CI<0.9), additivity (CI=0.9–1.1) or antagonism (CI>1.1). Drug target analysis was used for WiDr, H630 and SW1116 to investigate whether there was an increase in Pt-DNA adduct formation, DNA damage induction, cell cycle delay and apoptosis. Trifluorothymidine combined with OHP resulted in synergism for all cell lines (all CI<0.9). This was irrespective of schedule in which either one of the drugs was kept at a constant concentration (using variable drug ratio) or when the two drugs were added in a 1 : 1 IC_50_-based molar ratio. Synergism could be increased for WiDr using sequential drug treatment schedules. Trifluorothymidine increased Pt-DNA adduct formation significantly in H630 and SW1116 (14.4 and 99.1%, respectively; *P*<0.05). Platinum-DNA adducts were retained best in SW1116 in the presence of TFT. More DNA-strand breaks were induced in SW1116 and the combination increased DNA damage induction (>20%) compared with OHP alone. Exposure to the drugs induced a clear cell-cycle S-phase arrest, but was dose schedule and cell line dependent. Trifluorothymidine (TFT) and OHP both induced apoptosis, which increased significantly for WiDr and SW1116 after TFT–OHP exposure (18.8 and 20.6% respectively; *P*<0.05). The basal protein levels of ERCC1 DNA repair enzyme were not related to the DNA damage that was induced in the cell lines. In conclusion, the combination of TFT with the DNA synthesis inhibitor OHP induces synergism in colorectal cancer cells, but is dependent on the dose and treatment schedule used.

Most chemotherapy regimens used in the treatment of colorectal cancer consist of combinations of drugs to increase the therapeutic efficacy (Peters *et al*, 2000). The most common combinations include the fluoropyrimidine 5-fluorouracil (5FU) (Pinedo and Peters, 1988; Schmoll *et al*, 1999; Fishman and Wadler, 2001), together with irinotecan (CPT-11) and oxaliplatin (OHP) or more recently several targeted agents (Grivicich *et al*, 2001; Coutinho and Rocha Lima, 2003). For the platinum (Pt) analogue, OHP synergism with 5FU and leucovorin (LV) has been shown, whereas the drug shows little toxicity overlap and the combination has superior activity compared to 5FU/LV alone. Oxaliplatin is combined with 5FU/LV (FOLFOX and FLOX) regimens (Pelley, 2001; Andre and De Gramont, 2004; Wolmark *et al*, 2005), or in combination with the 5FU prodrug capecitabine (XELOX) (Cassidy *et al*, 2004) or CPT-11 (Goldberg *et al*, 2004).

Oxaliplatin is also active against other malignancies (Raymond *et al*, 1998c), and additive or synergistic effects with other anticancer drugs can be induced (Raymond *et al*, 2002). Oxaliplatin has distinct biochemical, pharmacological and cytotoxic properties compared with the related platinum compounds cisplatin and carboplatin (Rixe *et al*, 1996) and shows no crossresistance. All platinum compounds are cell cycle phase nonspecific and undergo nonenzymatic conversion to their active derivatives. They can react with DNA mainly by formation of Pt-DNA intrastrand adducts, and also by formation of interstrand and DNA–protein crosslinks, thereby blocking DNA replication and transcription (Raymond *et al*, 1998b; Woynarowski *et al*, 2000). Unlike the former two agents, OHP carries the diaminocyclohexane (DACH) ligand, which allows DNA lesions to avoid DNA mismatch repair and DNA damage recognition pathways (Fink *et al*, 1996; Vaisman *et al*, 1998). The formation of DACH-Pt-DNA adducts eventually results in DNA-strand breaks leading to the induction of apoptosis (Faivre *et al*, 2003; Arango *et al*, 2004). As 5FU resistance is regularly observed, other combinations enhancing OHP are of major interest to bypass 5FU resistance. Murakami *et al* (2000) previously showed that the fluoropyrimidine trifluorothymidine (TFT; trifluridine) was able to exert cytotoxicity against 5FU-resistant DLD-1 colon cancer cells.

Trifluorothymidine (TFT) is currently used as an antiviral agent (De Clercq, 2004) and is part of the novel oral antitumour drug preparation TAS-102 (Emura *et al*, 2004b; Tsuchiya *et al*, 2004; Temmink *et al*, 2005), which also consists of the antiangiogenic thymidine phosphorylase inhibitor TPI (Matsushita *et al*, 1999; Fukushima *et al*, 2000; Takao *et al*, 2000). TAS-102 is currently evaluated in different treatment schedules in phase I clinical trials (Hong *et al*, 2006). Trifluorothymidine acts by incorporation into DNA leading to DNA-strand breaks (Emura *et al*, 2004c), and by inhibition of thymidylate synthase (TS) (Eckstein *et al*, 1994; Temmink *et al*, 2004; Temmink *et al*, 2005), one of the major rate-limiting enzymes in DNA synthesis. In contrast to 5FU, TFT is not incorporated into RNA and does not need folates to form a ternary complex with TS. Trifluorothymidine is converted by thymidine kinase (TK) to 5-trifluoro-2′-deoxythymidine-5′-monophosphate (TF-TMP), which binds covalently to the active site of TS to inhibit its activity (Eckstein *et al*, 1994; Santi and Sakai, 1972). Thymidylate synthase inhibition results in an induction of a series of downstream events, eventually leading to cell death (Van Triest *et al*, 2000). These properties make TFT a suitable candidate to be combined with OHP, as both agents cause DNA damage and incorporation of TFT into DNA opposite to adenosine might affect the A-A-adduct formations.

In various colorectal cancer cell lines, we evaluated the interaction between the dual-targeted TFT and OHP in relation to formation of Pt-DNA adducts and the induction of DNA-strand breaks, cell cycle delay and apoptosis.

## MATERIALS AND METHODS

### Materials

Dulbecco's modified Eagle's medium (DMEM) and Hepes buffer were purchased from Cambrex BioScience (Verviers, Belgium) and foetal bovine serum (FBS) from Greiner Bio-One (Frickenhausen, Germany). Trifluorothymidine (TFT) was synthesised and provided by Taiho Pharmaceuticals Co. (Tokushima, Japan). Oxaliplatin was a gift from Sanofi-Synthelabo Inc. (Lyon, France). Sulforhodamine B (SRB) protein dye and propidium iodide (PI) were purchased from Sigma-Aldrich Chemicals (Zwijndrecht, The Netherlands). Bovine serum albumin (BSA) was obtained from Merck (Darmstadt, Germany). The PVDF membranes, ECL Hyperfilms and ECL Plus detection kit were obtained from Amersham Biosciences (Buckinghamshire, UK). The primary antibodies mouse-anti-human MLH1, MSH2, ERCC1 and *β*-tubulin were purchased from Zymed Lab Inc. (San Francisco, CA, USA), Oncogene Research Products (Cambridge, MA, USA), NeoMarkers Inc. (Fremont, CA, USA) and BD Biosciences (San Jose, CA, USA), respectively. The secondary peroxidase-conjugated antibodies were purchased from DAKO (Glostrup, Denmark). All other chemicals were of analytical grade and commercially available.

### Cell culture

The colorectal cancer cell lines WiDr, H630, Colo320, SNU-C4 and SW1116 were used in this study. WiDr, Colo320 and SW1116 were obtained from the American Type Culture Collection (ATCC); H630 and SNU-C4 were a kind gift from Dr PG Johnston (at that time at the National Cancer Institute, Bethesda, MD, USA). All these cell lines were cultured in DMEM supplemented with 10% heat-inactivated FBS and 20 mM Hepes buffer (without antibiotics). They were grown as adherent monolayers in a humidified atmosphere containing 5% CO_2_ at 37°C and were maintained in exponential growth. All cell lines except SNU-C4 are p53 mutated where no or decreased induction of p53 expression is present (Peters *et al*, 2002).

### Growth inhibition experiments and multiple drug-effect analysis

To assess cytotoxicity of the cell lines to the drugs the SRB cytotoxicity assay was used (Skehan *et al*, 1990; Keepers *et al*, 1991). In brief, the cells were seeded in 100 *μ*l medium in triplicate in 96-well flat-bottom plates (Greiner Bio-One, Frickenhausen, Germany) in different densities depending on their growth rate (5000–10 000 cells well^−1^). After 24 h, 100 *μ*l drug containing medium was added to the wells and the cells were incubated for another 72 h. Thereafter, cells were fixed using trichloro-acetic acid (TCA) and stained with the SRB dye. Differences in optical density (OD measured at 540 nm) between the treated cells and untreated control cells were compared. The percentage of control growth at the start and end of drug exposure was calculated using the formula: [(OD_treated_/OD_start drug exposure_)−1]/[(OD_control_/OD_start drug exposure_)−1] × 100%. The obtained IC_25_ and IC_50_ values were expressed as the concentrations that corresponded to a reduction of cellular growth by 25 and 50%, respectively, when compared with values of the untreated control cells.

All cell lines were exposed to the drugs alone or in combination. Different combination variants were used to test the interaction of the drugs: either one drug was added at a concentration that caused 25% growth inhibition and the other drug was added in a concentration range, or both drugs were added in a fixed IC_50_-based molar ratio. To evaluate the effect of a combination of two drugs, CalcuSyn (Version 1.1.1 1996, Biosoft, Cambridge, UK) was used, a program based on the method of Chou and Talalay (Chou and Talalay, 1984). The multiple-drug effect analysis provides a numerical, statistically evaluable method, and provides classical isobolograms which give information on synergism, but no quantification.

The absorbance values of drug treated wells were compared to the absorbance values of the control wells to calculate each fraction affected (FA), where FA=0.25 means a decrease in absorbance and growth of 25%. From the median drug–effect plots the dose that reduced absorbance by 50% (*D*_x_) and the slope were calculated, after which the doses of the separate drugs and combination required to induce various levels of cytotoxicity were calculated (Temmink *et al*, 2006). For each level of cytotoxicity, a mutually nonexclusive combination index (CI) was calculated using the formula CI=[(*D*)_1_/(*D*_*1−*FA_)_1_]+[(*D*)_2_/(*D*_*1−*FA_)_2_]+[α(*D*)_1_(*D*)_2_/(*D*_*1−*FA_)_1_(*D*_*1−*FA_)_2_]. The parameters (*D*)_1_ and (*D*)_2_ represent the doses of the combination of drugs in a fixed ratio, whereas (*D*_*1−FA*_)_1_ and (*D*_*1−FA*_)_2_ are the doses of the individual drugs resulting in the effect 1−FA (*α*=1 for mutually nonexclusive drugs). The CI indicates synergism (CI<0.9), additivity (CI=0.9–1.1) or antagonism (CI>1.1). A mean CI was calculated from data points with FA>0.5 for the combinations in which one drug was added at a constant concentration, and from the FA values 0.6, 0.75 and 0.9 for the fixed ratio combinations. The FA range used to calculate the average CI values in the combination experiments did not include CI values of FA<0.5, which was considered as not relevant growth inhibition (Peters *et al*, 2000), because one aims to achieve the maximal effect of the drugs tested on cancer cells. Furthermore, CI values at FA>0.9 were not taken into account owing to higher chance to subject to error, and because this would not add much more information. Averaging the CI values for the whole FA=01–0.9 range might also lead to false interpretation of the data, and different effects were seen at different FA values (Figure 2B).

### Determination of platinum-DNA adduct formation

The formation of Pt-DNA adducts into DNA after exposure of cells to OHP was measured using a protocol adapted from Van Moorsel *et al* (1999). Cells were grown to 70–80% confluence in 75 cm^2^ tissue culture flasks (Greiner Bio-One) and then treated with 200 *μ*M OHP for 24 h at 37°C, either alone or in combination with 10 *μ*M TFT (4 h preincubation or/and simultaneous exposure), to investigate whether TFT influenced Pt-DNA adduct formation induced by OHP. Thereafter, the cells were either harvested or cultured in drug-free medium for another 3 h. After washing with phosphate-buffered saline (PBS), trypsinising and harvesting the cells, the cell pellets (at least 5 × 10^6^ cells) were resuspended in ice-cold DNA STAT-60 lysis reagent (Tel-Test Inc., Friendswood, TX, USA; 5 × 10^6^ cells 1 ml^−1^). The DNA extraction, precipitation and washing steps were carried out according to the protocol of the manufacturer. The obtained DNA pellets were dissolved in a TE buffer (10 mM Tris, 1 mM EDTA; pH 8.0). DNA content was estimated by measuring optical densities with the NanoDrop apparatus (NanoDrop Technologies Inc., Wilmington, DE, USA). All samples had an OD_260_/OD_280_ ratio>1.9 indicating uncontaminated DNA. Sodium chloride (1.65 M) was added in a volume of 0.1 to the dissolved DNA. A calibration curve was made using different solutions of OHP (0–2 *μ*M). The Pt content of the samples and standards (expressed as pmol *μ*g^−1^ DNA) was measured using Atomic Absorption Spectrometry (AAS).

### Comet DNA damage assay

The alkaline comet assay or single-cell gel electrophoresis assay was performed to detect DNA-strand breaks associated with DNA damage (Olive and Banath, 1997; Olive, 2002). The assay is based on the alkaline lysis of labile DNA at damaged sites where denatured and cleaved DNA fragments migrate easily out of the cell under the influence of an electric field than undamaged DNA. To study the role of DNA damage in the interaction between OHP and TFT, we exposed 1.6 × 10^5^ cells in six-well plates to IC_90_ of the drugs for 24 h. This concentration caused 90% growth inhibition in the 72 h growth inhibition studies. As positive controls, cells were exposed to 100 *μ*M H_2_O_2_ for 20 min at 4°C to induce significant oxidative damage. Untreated cells were used as negative controls. The cell suspensions consisted of 8 × 10^4^ cells ml^−1^ in PBS. The lysis, electrophoresis and staining procedures were previously described (Hartley *et al*, 1999).

After the PI staining procedure, the slides were rinsed in distilled water and viewed within 24 h. The DNA from the cells was visualised using a Zeiss Axioskop2 fluorescence microscope (Carl Zeiss Mikroscopie, Jena, Germany) with a Zeiss HBO-103W/2 mercury light source (using 546 nm light excitation filter). The microscope was attached to a Zeiss AxioCam MRm Camera using an Achroplan × 20 objective. The obtained comet images were analysed using Scion Image software (Scion Corp., Frederick, MD, USA). DNA damage was quantified as the tail moment, the product of the percentage of total DNA in the comet tail and the mean distance between the head and tail distributions (Hartley *et al*, 1999). At least 20 comets were analysed per slide.

### Apoptosis analysis

The terminal deoxynucleotidyl transferase (-TdT-)mediated dNTP-labelling (TUNEL) method was used for the detection of cells undergoing apoptosis. For this purpose we used the TdT-DNA-Fragment End Labeling Kit (FragEL™; Calbiochem, Oncogene Research Products, Cambridge, MA, USA). In this method, TdT binds to exposed 3′-OH ends of DNA fragments generated in apoptotic cells in order to add biotin-(un)labeled dNTPs, which are detected using a streptavidin–horseradish peroxidase conjugate. Cells were plated into 6-wells plates as described in the cell cycle analysis section and were exposed 48 h or 72 h to IC_75_ of OHP or/and TFT (with or without a 24 h TFT preincubation period). After the incubation period cytospin slides were made using a formaldehyde fixation protocol. The staining was performed according to the manufacturer's recommended procedure. Cells stained positive with 3′-3′-diaminobenzidine (DAB) produce a brownish colour, whereas nonreactive cells were counterstained with methylgreen. Actinomycin D-treated HL60 cells were included in the kit and served as positive controls. Negative controls were cytospins of untreated cells in their logarithmic growth phase. Using light microscopy, 1000 cells were counted twice for positive/negative staining on randomly selected areas on the glass slide, and the apoptotic index was calculated as the percentage of positive staining cells. Cells were defined as apoptotic when the (major part of) nuclear area was DAB-labelled.

### Cell cycle analysis

Cell cycle distribution was measured on cells exposed to the drugs alone or in combination (Cloos *et al*, 2002). On day 0, a series of six-well plates were filled with 2 ml cell suspensions at a concentration of 2 × 10^5^ cells well^−1^. After 24 h incubation, cells were exposed 48 h to IC_50_ or IC_75_ concentrations of the drugs alone or in combination (with or without a 4 h TFT preincubation period). Pilot experiments demonstrated that the optimal exposure time was 48 h. The percentage of cells in the different cell cycle phases (G_1_, S and G_2_M) was measured using FACScan (Becton Dickinson Immunocytometry Systems, San Jose, CA, USA). For each measurement, 20 000 cells were counted, and each sample was assayed in duplicate. For calculation of the cell cycle distribution, the Becton Dickinson's CellQuest software was used.

### Western blot analysis

Frozen pellets of the cell lines were lysed in buffer (0.1% Triton X-100, 150 mM NaCl, 10 mM Tris-HCl, 50 mM *β*-glycerophosphate; pH 7.5) and sonificated 5 × 5 s with 5 s intervals. After centrifugation (10 min 13 000 **g** at 4°C), the supernatants were measured for protein content using the BioRad Bradford protein assay (Bradford, 1976). For determination of protein expression, 20 *μ*g of total protein from each cell line was loaded and separated on a 10% SDS-PAGE gel, followed by blotting on a PVDF membrane. Each membrane was preincubated O/N at 4°C with blocking buffer (5% milk powder in 0.15 M NaCl, 0.05% Tween-20, 10 mM Tris-HCl (TBS-T); pH 8.0) to prevent aspecific antibody binding. This was followed by 2 h incubation at room temperature with the primary antibodies diluted in TBS-T containing 5% BSA. After three 10 min washing steps with TBS-T, the blots were incubated for 1 h at room temperature with the secondary antibodies diluted in TBS-T containing 2% milk powder. Detection of antibody binding was measured with enhanced chemoluminescence (ECL Plus detection solution and ECL Hyperfilms). Protein levels were determined by densitometric scanning.

### Statistical evaluation

The (un)paired Student's *t*-test was used for statistical evaluation of the results. Changes were considered to be significant when *P*<0.05.

## RESULTS

### Evaluation of the combination OHP with TFT

The sensitivities of the colorectal cancer cell lines for OHP and TFT varied considerably (Table 1). SNU-C4 was most sensitive to OHP; H630 and Colo320 were most sensitive to TFT. WiDr was most resistant to OHP, whereas SW1116 was the most TFT-resistant cell line. The obtained IC_50_ values were also used to calculate the drug ratios for the fixed ratio combinations.

**Figure 1:**
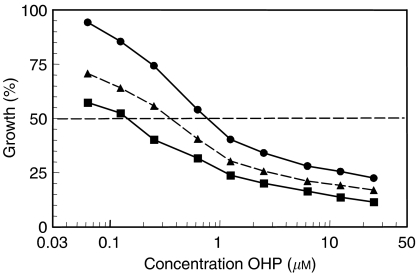
Representative growth inhibition curves for the combination OHP-TFT using a variable drug ratio. SW1116 cells were exposed 72 h to OHP, TFT or OHP+TFT in which OHP was added at various concentrations (OHPv) and TFT was kept at a constant IC_25_ concentration (TFTc). Filled circles and solid line, OHPv; filled triangles and broken line, expected growth inhibition calculated from the single drug doses; filled squares and solid line, OHPv+TFTc. The growth inhibition curves were obtained from three separate experiments. Per experiment, an average CI value was calculated from all data points with FA>0.5. In this example, mean CI=0.64. All s.e.m. values <10%.

**Figure 2:**
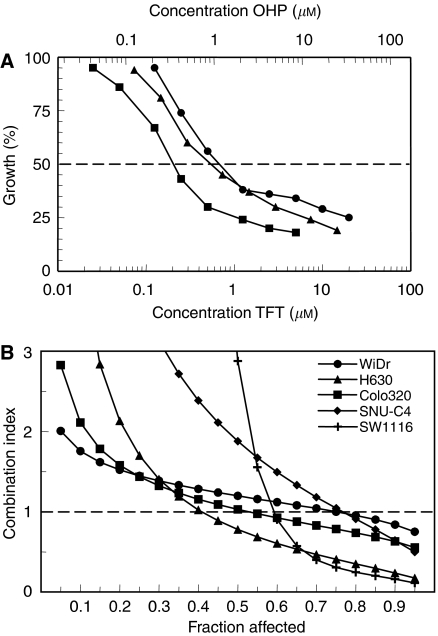
Illustrative growth inhibition curves (**A**) and FA-CI plots (**B**) for the combination OHP-TFT using a fixed drug ratio. Figure A: H630 cells were exposed 72 h to TFT (filled circles), OHP (filled triangles) or TFT+OHP (filled squares). Figure B: Average CI values calculated from each FA for all five cell lines used in the study. Per experiment, an average CI value was calculated from data-points with FA=0.6, 0.75 and 0.9. All s.e.m. values of data points <15%.

**Figure 3:**
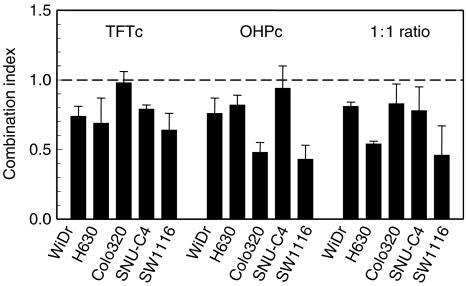
Synergy analysis of OHP combined with TFT for the colorectal cancer cell lines. Interpretation of CI values: CI<0.9 means synergism; CI=0.9–1.1 means additive; CI>1.1 means antagonism. TFTc: combination of the drugs in which TFT was kept at a constant concentration (IC_25_); OHPc: combination of the drugs in which OHP was kept at a constant concentration (IC_25_); 1:1 ratio: the two drugs were added in a fixed 1:1 IC_50_-based molar ratio. A mean CI was calculated from data points with FA>0.5 for the combinations with variable drug ratio, and from the FA values 0.6, 0.75, 0.9 for the fixed ratio combinations. Values (mean CI±s.e.m.) are based on 3–4 separate experiments. See also Materials and Methods.

**Figure 4:**
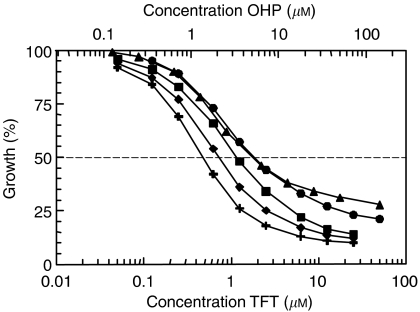
Representative growth inhibition curves for simultaneous and sequential OHP-TFT combinations using a fixed drug ratio. WiDr cells were exposed 72 h to OHP with or without TFT in different schedules: TFT alone (filled circles), OHP (filled triangles), TFT+OHP (filled squares), 24 h TFT alone >48 h TFT+OHP (filled diamonds), 24 h OHP alone >48 h TFT+OHP (filled crosses). The growth inhibition curves were obtained from 3–5 separate experiments (all s.e.m. values <15%). Per experiment an average CI value was calculated from all data points with FA>0.5.

Multiple drug-effect analysis was performed for the combinations of TFT with OHP using the CalcuSyn software. Figure 1 shows representative growth inhibition curves with SW1116 cells for the combinations using a variable concentration of OHP and a fixed concentration of TFT, illustrating at least additive cytotoxicity. Dose–effect and FA-CI plots illustrating the effects of TFT–OHP fixed drug ratio combinations are depicted in Figure 2. Figure 2A depicts growth inhibition curves of H630 cells exposed to OHP and TFT at various concentrations based on a 1 : 1 IC_50_ ratio, and clearly shows that the combination causes more growth inhibition than each drug alone. To indicate the effects at different FA values average CI values were calculated for each FA. Figure 2B shows the mathematical analysis of these data and demonstrates synergism at FA>0.5 for H630, as well as the other cell lines. The FA values were obtained after exposure of cells to a series of drug concentrations of TFT and/or OHP, depending on the combination variant. The mean CI values of the fixed ratio TFT–OHP combination variants are shown in Figure 3. The effects of these combinations were synergistic in the cell lines (all CI < 0.9). For the variable drug ratio combinations, only in Colo320 (TFTc; mean CI=0.98±0.08) and SNU-C4 (OHPc; mean CI=0.94±0.16) the interaction between the drugs was not more than additive. The extent of synergism differed according to the cell line and combination variant. Strongest synergism was observed with SW1116 cells for all three combination variants (0.43⩽mean CI⩽0.64). For further evaluation of the TFT–OHP combination, only the cell lines WiDr, H630 and SW1116 were used, because for these cell lines large differences in drug sensitivity were found. Incubating these cells the first 24 h with only TFT or OHP decreased synergism for the 1 : 1 ratio combinations for H630 and SW1116 (0.6⩽mean CI⩽1.2; data not shown), but not for WiDr, where increased synergism was observed for both sequential combinations (Figure 4); TFT preincubation decreased the mean CI to 0.6 compared to the simultaneous combination (mean CI=0.81±0.06), whereas OHP preincubation decreased the mean CI to 0.4.

### Formation of Pt-DNA adducts

**Figure 5:**
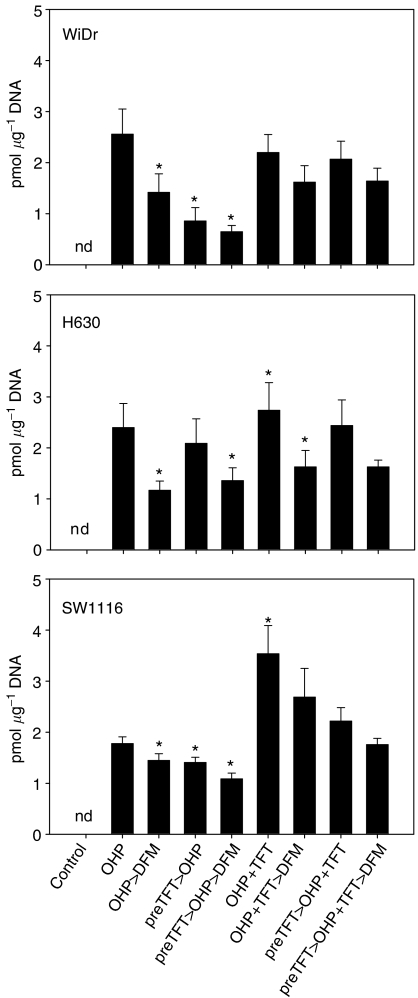
Pt-Adduct formation in DNA after exposure of WiDr, H630 and SW1116 cells to OHP with or without TFT in different schedules. The cell lines were exposed 24 h to 200 *μ*M OHP alone or with 10 *μ*M TFT, either simultaneously and/or with a 4 h preincubation period (preTFT). To determine retention of the formed Pt adducts the cells were incubated an additional 3 h in drug-free medium (DFM). Values are Means±s.e.m. (*n*=4). ^*^P<0.05 compared to 24 h OHP. nd=nothing detected.

Because the synergism might be related to an influence of TFT on OHP-induced DNA damage by affecting the accumulation of Pt-DNA adducts, we studied the formation and retention of Pt-DNA adducts in three cell lines. The cell lines were selected, because one is rather insensitive to OHP (WiDr) or TFT (SW1116) or sensitive to both drugs (H630). As WiDr and SW1116 have an intermediate sensitivity to the other drugs, we reasoned that this panel would be representative for all five cell lines, also because the synergism patterns were similar. The measurements were limited to Pt-DNA adduct formation. Total uptake of OHP in cells was not measured, as it was considered unlikely that a nucleoside analogue would affect uptake of a platinum compound into a cell, because transport mechanisms for platinum analogs are not affected by nucleoside analogs, but Pt-DNA adduct formation and repair are (Bergman *et al*, 1996; Van Moorsel *et al*, 1999; Yang *et al*, 2000). With OHP alone adduct formation ranged from 1.8 to 2.6 pmol *μ*g^−1^ DNA (Figure 5). Pretreatment with 10 *μ*M TFT decreased the formation of Pt-DNA adducts by 13% or more. Simultaneous treatment resulted in a significant increase in the formation of Pt-DNA adducts in H630 and SW1116 (14.4 and 99.1%, respectively; *P*<0.05), but not in WiDr. However, TFT pretreatment decreased the Pt-DNA adduct formation (>6%). The formed Pt-DNA adducts after exposure to OHP alone were best retained in the SW1116 cells (81.6%) compared with H630 and WiDr (< 56%), and TFT treatment increased this in the latter two cell lines (>10%).

### Formation of DNA-strand breaks and induction of apoptosis

**Figure 6:**
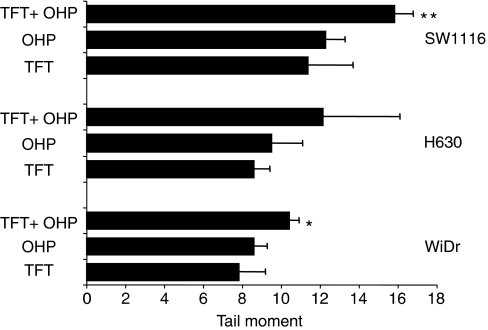
DNA damage induction in WiDr, H630 and SW1116 cells using the Comet assay. The cells were exposed 24 h to [IC_90_] OHP or [IC_90_] TFT alone or in combination. These concentrations were: WiDr: 75 *μ*M TFT, 75 *μ*M OHP; H630: 50 *μ*M TFT, 30 *μ*M OHP and SW1116: 200 *μ*M TFT, 80 *μ*M OHP. Values are means±s.e.m. (*n*=3). Significant differences compared with OHP alone: ^*^P<0.05; ^**^P<0.01 (paired *t*-test).

Using the comet assay, we evaluated whether more DNA damage was induced in the cells after 24 h exposure to a combination of TFT–OHP at their IC_90_ concentrations compared to each drug alone (Figure 6). The WiDr, H630 and SW1116 cell lines were used because the combinations induced synergistic cytotoxicity in these cells. Relatively high concentrations of the drugs were chosen to determine the potency of this combination to induce DNA damage in the cells. The calculated tail moment represents DNA-strand break formation induced by the drugs. At equally growth inhibitory concentrations there was consistently more DNA damage in the cells after exposure to OHP compared to TFT, although not significantly (*P*>0.05). About 20–30% more DNA damage was induced after exposure to TFT–OHP compared to OHP alone. Most DNA-strand breaks were induced in SW1116 cells (mean tail moment of 15.8±0.5). Comparable results were seen after exposure to IC_75_ concentrations of the drugs (all mean tail moments < 10; data not shown).

Table 2 summarises the results of apoptosis induction using the TUNEL apoptosis assay when the cells were exposed 72 h to IC_75_ concentrations of the drugs. In the control samples, less than 3% apoptosis was found in the total cell population. When the cells were exposed to OHP alone or TFT alone, about equal amounts of apoptosis were induced (range 6.7–16.2% apoptotic cells). This was at least 3.7-fold higher compared with the controls. After TFT–OHP drug treatment most apoptosis was induced in the WiDr and SW1116 cell populations (18.8 and 20.6%, respectively), with less in H630 cells; in each cell line, TFT–OHP induced significantly more apoptosis compared with OHP alone (*P*<0.05). Shortening the total exposure to OHP by 24 h decreased the induction of apoptosis by at least 30% for all cell lines. A TFT pre-exposure period of 24 h, followed by 48 h OHP exposure, increased apoptosis induction slightly compared to 48 h OHP exposure, but this was still significantly lower compared with 72 h TFT–OHP exposure (at least 45%; *P*<0.01). Induction of apoptosis was generally in agreement with the results obtained from the DNA damage experiments.

### Induction of cell cycle arrest

The combination TFT–OHP was also evaluated using flow cytometry analysis to determine the cell cycle distribution after 48 h drug(s) exposure (Table 3). This time point was chosen because at 48 h substantial changes can be seen in cell cycle distribution, which are not yet observed after 24 h exposure. Trifluorothymidine induced an S-phase arrest, or G_2_M-phase arrest, although this was concentration-, schedule- and cell line-dependent. In all cell lines TFT or OHP induced a clear reduction in the G_1_-phase cell population, probably owing to DNA synthesis inhibition. This was more pronounced after TFT exposure than after OHP exposure. For all three cell lines a significant increase in the S phase cell population was seen after exposure to TFT or OHP alone (6.2–44.7%; *P*<0.05). Only in WiDr cells TFT induced a clear S-phase arrest whereas OHP induced a G_2_M-phase arrest (both>50%). The combination TFT–OHP induced a strong S-phase arrest for all cell lines (*P*<0.01). Compared to OHP exposure, the combination significantly increased the S-phase cell population for WiDr and H630, in contrast to SW1116, where TFT did not increase (%) cells in the S-phase, but (%) cells in G_2_M phases was increased from 27.8% to 38.1% (*P*<0.05). The G_1_ population was also more decreased (⩾6.2%). A 4 h TFT preincubation period hardly affected cell cycle distribution induced by OHP, except for SW1116, where G_2_M population increased from 27.8 to 51.3%; (*P*<0.01). Surprisingly, this result was not observed in the growth inhibition and apoptosis experiments. Comparable results were obtained when cells were exposed to IC_50_ concentrations of the drugs, but induction of S-phase arrest was less potent (data not shown).

### Protein levels of DNA repair enzymes

**Figure 7:**
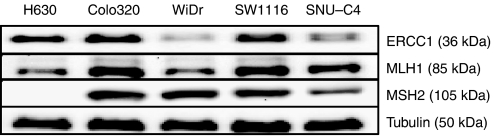
Basal protein levels of the DNA repair enzymes ERCC1, MLH1 and MSH2 for the colorectal cancer cell lines H630, Colo320, WiDr, SW1116 and SNU-C4. Equal protein amounts from unexposed cells were used for Western blotting as described in the Materials and Methods section.

Figure 7 shows the basal expression of ERCC1, MLH1 and MSH2 DNA repair proteins for the colorectal cancer cell lines. There was no direct correlation with OHP or TFT sensitivity. For example, SW1116 is more sensitive to the drug combination compared to WiDr, inducing significantly more DNA-strand breaks in these cells, but also has higher ERCC1 levels, which is part of the DNA excision repair system, which is mainly involved in the repair of OHP-induced DNA damage.

## DISCUSSION

In the present study we demonstrated synergism between OHP and TFT, which was found for drug combinations using either variable or fixed drug ratios. The most pronounced effects were seen in SW1116 cells, where strong synergism was induced at high effect levels. There is a parallel enhancement of OHP-induced DNA damage and apoptosis by TFT. We have demonstrated that this damage may be explained by increased formation of Pt-DNA adducts, which possibly is related to increase incorporation of TFT into the DNA.

The platinum compound OHP is currently routinely used in the treatment of colorectal cancer patients in combination with 5FU (or its prodrug capecitabine). In the case of 5FU resistance, it may also be combined with irinotecan (CPT-11), thereby improving response and survival rates (Maindrault-Goebel *et al*, 1999; Coutinho and Rocha Lima, 2003; Andre and De Gramont, 2004; Smorenburg *et al*, 2006). Oxaliplatin acts by effectively disrupting the DNA replication and transcription in the cell, but the downstream molecular events underlying its mechanism of action have not been characterised very well (Arango *et al*, 2004). Oxaliplatin (OHP) itself reduces growth of the cells at clinically relevant concentrations, which can be associated with a G_2_M-phase arrest in the cell cycle (Raymond *et al*, 2002) and increased induction of apoptosis (Tassone *et al*, 2002). Inducing cell death by chemotherapeutics is an important terminal mechanism to eliminate malignant cells *in vivo* (Broker *et al*, 2005). Trifluorothymidine was able to enhance OHP-induced apoptotic cell death, possibly owing to extensive DNA damage induction by increased Pt-DNA adduct formation.

In preclinical studies using colorectal cancer cells, OHP has been combined with a variety of anticancer metabolites to improve its killing efficacy, such as TS inhibitors (Raymond *et al*, 1997), taxanes and topoisomerase I inhibitors (Raymond *et al*, 1998a; Raymond *et al*, 2002). Trifluorothymidine, in the form of the orally administered TAS-102 formulation, might be a good alterntive to 5FU in the combination with OHP. Trifluorothymidine incorporation into DNA also induces DNA-strand breaks, and as a result of intracellular thymidine depletion owing to inhibition of TS, misincorporation of uracil into DNA occurs, thereby enhancing the induction of DNA damage (Webley *et al*, 2001). Trifluorothymidine itself is a dual-targeted agent, and the other part of TAS-102 is TPI, which is a potent TP inhibitor (*K*_*i*_=0.17 nM) (Fukushima *et al*, 2000). *In vivo*, TFT is very dependent on TP activity, since adding TPI enhances bioavailability of TFT (Emura *et al*, 2005), which may favour the combination OHP-TFT, possibly leading to more DNA damage in cells and higher cytotoxicity. Secondly, TP is also known as PD-ECGF (Moghaddam and Bicknell, 1992; Sumizawa *et al*, 1993), which has stimulating properties on blood vessel formation in solid tumours (Takahashi *et al*, 1998). Previous studies showed that TPI inhibits blood vessel formation, thereby increasing apoptosis (Matsushita *et al*, 1999) and suppressing the formation of metastases (Takao *et al*, 2000). In contrast to 5FU-based formulations, the orally given TAS-102 has both cytotoxic and antiangiogenic properties.

Trifluorothymidine and 5FU are both potent TS inhibitors, but in contrast to TFT, 5FU is also incorporated into the RNA of cancer cells (Peters, 2002). Furthermore, TFT can only be activated by TK, thus decreased TK activity will affect TFT conversion to TF-TMP, and subsequently leads to decreased TS inhibition and TF-TTP incorporation into the DNA (Temmink *et al*, 2005). 5-fluorouracil can be anabolised to its active forms in two alternative pathways next to TK activation by uridine phosphorylase or orotate phosphoribosyltransferase, which seem the most important pathways (Peters, 2002). On the other hand, TFT is activated rapidly because it only needs one phosphorylation step in contrast to 5FU, and therefore TFT can be active against 5FU-resistant colorectal cancer cells (Murakami *et al*, 2000; Emura *et al*, 2004a). Thus, compared with 5FU, TFT is clearly a different drug than 5FU leading to different drug interactions. In general, 5FU combinations with cisplatin show variable interactions (Van der Wilt *et al*, 1992), whereas combinations of 5FU with OHP in general lead to synergistic interactions (Fischel *et al*, 1998). Our data clearly show synergism of TFT with OHP in all conditions.

Our data allow some preliminary conclusions about appropriate scheduling of OHP and TFT. Ideally, a combination should be at least additive in its anticancer effects but without excessive normal tissue toxicity. Oxaliplatin is administered by infusion, and in animals TAS-102 is orally administered and is most effective when used as a multiple daily dosing schedule (three times daily at 150 mg kg^−1^ day^−1^) (Emura *et al*, 2004b, 2004c). In human tumour xenografts in mice incorporation of TFT into DNA was significantly higher with divided dosing than single dosing, resulting in enhanced antitumour activity and no additional side effects. Regarding the TFT–OHP combination, we observed that preincubation with TFT resulted in less formation of Pt-DNA adducts and less subsequent induction of apoptosis than did simultaneous incubation without preincubation. The exact mechanism responsible for this decrease in formation of Pt-DNA adducts remains to be elucidated. Nonetheless, our data suggest that simultaneous incubation is ideal to maximise anti-tumour effects.

We studied both fixed and variable drug concentration ratios of TFT and OHP. Previous studies of drug combinations have often focused on a fixed ratio between the drug concentrations, but a fixed ratio does usually not reflect the actual ratio in patients (Van Moorsel *et al*, 1999; Temmink *et al*, 2006). Oxaliplatin and TFT show comparable growth inhibition curves (Figures 1 and 2), indicating the same growth inhibition kinetics. The synergism was observed at FA>0.5. Although data at low drug concentrations suggest antagonism, we do not consider this part of the curve as relevant, since one aims to expose tumour cells to the highest possible cytotoxic drug concentration; this means that 25% growth inhibition is irrelevant, because this would mean that the tumour cell population still grows at 75% compared to untreated tumour cells. Our data are favourable for potential future application of this combination since synergism could be induced for both combination variants (fixed and variable ratios) for most cell lines. Trifluorothymidine has a *t*_1/2_ of about 15 min, but administration of several doses of TPI together with divided dosing of TFT increases the AUC and *t*_1/2_ dramatically, which in turn could increase OHP-induced cytotoxicity when given simultaneously within the same time period. OHP is given by infusion and has a *t*_1/2_ of less than 30 min (Ehrsson *et al*, 2002). This will result in a variable ratio, and therefore the variable ratio used in our experiments will better reflect a clinical situation.

Effects of combined OHP and TFT were cell line-dependent. The most significant results were obtained with the SW1116 cell line, which is the cell line most resistant to TFT. This is a promising perspective since combinations are aimed to reduce or bypass resistance. Trifluorothymidine enhanced OHP-induced cytotoxicity by increasng of the formation of Pt-DNA adducts (almost two-fold), which were well retained. This was accompanied by enhanced DNA-strand break formation, cell cycle arrest and subsequent apoptotic cell death, possibly mediated through caspase-3. Possibly because H630 is far more sensitive to both TFT and OHP than SW1116, the drug combination showed less pronounced effects compared with SW1116. The present study shows that no clear mechanism can be associated with the sensitivity to OHP or to the synergism between OHP and TFT. Platinum-DNA (Pt-DNA) adduct formation is enhanced, as well as DNA damage. However, with the comet assay it is difficult to assess the real tail moment values below five, and as it is not justified to add up the tail moments , one can only conclude that DNA damage is higher in the combination and is highest in the most synergistic cell line SW1116. In addition, on this cell line, the most pronounced changes in cell cycle distribution (increased G_2_M) were found, possibly adding to the synergistic effect. This might be due to differences in TS levels or the incorporation rate of TFT in the cells. The present studies were also performed to see whether DNA damage repair was of any influence. The Western blot results showed that the protein expression levels of ERCC1, MLH1 and MSH2 DNA repair proteins do not correlate with the observed DNA damage induced by TFT–OHP. This was expected for MLH1 and MSH2 (DNA mismatch repair), which are mainly involved in the repair of cisplatin-induced DNA damage, not OHP-induced DNA damage. A possible increase of TFT incorporation into DNA by OHP might also interfere with the repair of OHP-induced DNA damage by ERCC1.

In conclusion, the combination of TFT with OHP is synergistic in colorectal cancer cells, which is related to increased Pt-DNA adduct formation and retention, cell cycle arrest and DNA damage induction. Therefore, our *in vitro* results provide a rationale for a clinical study of TAS-102 (TFT+TPI) together with OHP, with a potential value in the (second-line) treatment of colorectal cancer patients. Furthermore, the approval of the biological agents bevacizumab (Avastin®) and cetuximab (Erbitux®) might also be of interest for potential TAS-102-involving combinations, thereby enhancing inhibition of angiogenesis or targeting EGFR, respectively.

## Figures and Tables

**Table 1:** Growth inhibition of TFT and OHP for the colorectal cancer cell lines

**Cell line**	**TFT**	**OHP**
WiDr	2025±527^a^	6000±577
H630	453±114^a^	773±37
Colo320	533±133^a^	2083±309
SNU-C4	830±214	308±4
SW1116	7450±1340	1128±189

Cells were exposed to a concentration range of TFT or OHP for 72 h. Values (IC_50_ in nM) are means±s.e.m. of 3–5 experiments

a Results are partly published (Temmink *et al*, 2005).

**Table 2:** Induction of apoptosis by OHP, TFT or both drugs in WiDr, H630 and SW1116 cells

	**Cell line**		
**Drug Treatment**	**WiDr**	**H630**	**SW1116**
Control	2.3±0.6^**^	1.6±0.3^**^	2.9±0.2^**^
TFT IC_75_ 72 h	16.2±2.5	6.7±0.5^**^	10.6±1.7
OHP IC_75_ 72 h	13.2±1.7	8.2±3.0	13.7±2.2
OHP IC_75_+TFT IC_75_ 72 h	18.8±3.1^*^	13.1±1.0^*^	20.6±1.5^**^
OHP IC_75_ 48 h alone	8.9±1.2^*^	3.5±0.3^**^	7.0±0.7^**^
TFT IC_75_ 24 h>OHP IC_75_ 48 h	9.3±1.1^*^	7.0±0.7^*^	9.0±0.9^*^

The cells were exposed 72 h to [IC_75_] of the drugs, either alone or in combination. These concentrations were: WiDr: 25 *μ*M TFT, 10 *μ*M OHP; H630: 10 *μ*M TFT, 3.5 *μ*M OHP and SW1116: 100 *μ*M TFT, 10 *μ*M OHP. Values (%) represent [apoptosis population]/[total cell population] and are expressed as means±s.e.m. (*n*=3). Compared to OHP alone: ^*^P<0.05; ^**^P<0.01 ((un)paired *t*-test).

**Table 3:** Cell cycle distribution of WiDr, H630 and SW1116 cells following OHP and/or TFT exposure

	**Cell cycle distribution**		
**Drug treatment**	**(%) G1**	**(%) S**	**(%) G2M**
*WDr*			
Control	46.8±3.0	15.2±0.9	38.0±2.9
OHP IC_75_ 48 h	25.8±2.6	21.4±1.8	52.8±3.9
TFT IC_75_ 48 h	3.4±0.6^**^	59.9±3.4^**^	36.7±3.1^*^
TFT IC_75_+OHP IC_75_ 48 h	18.1±4.1	53.4±6.0^**^	28.5±3.1^**^
preTFT>OHP IC_75_ 48 h	30.3±1.0	25.4±4.3	44.3±5.2
preTFT>TFT IC_75_+OHP IC_75_ 48 h	6.4±2.0^**^	55.2±4.9^**^	38.4±3.3^*^
			
*H630*			
Control	38.9±1.2	24.2±0.9	36.9±2.0
OHP IC_75_ 48 h	19.8±7.9	37.1±3.2	43.1±5.0
TFT IC_75_ 48 h	12.1±5.7	43.9±7.1	44.1±5.9
TFT IC_75_+OHP IC_75_ 48 h	13.6±3.5	49.1±2.6^*^	37.3±0.9
preTFT>OHP IC_75_ 48 h	26.0±1.4	30.6±1.9	43.4±2.9
preTFT>TFT IC_75_+OHP IC_75_ 48 h	11.5±3.7	52.7±0.8^**^	35.8±3.5
			
*SW1116*			
Control	46.9±2.6	23.2±2.0	29.9±0.7
OHP IC_75_ 48 h	30.5±1.9	41.7±3.0	27.8±1.6
TFT IC_75_ 48 h	8.5±1.1^**^	35.8±2.5	55.7±2.1^**^
TFT IC_75_+OHP IC_75_ 48 h	12.7±1.2^**^	49.2±2.2	38.1±2.6^*^
preTFT>OHP IC_75_ 48 h	12.6±0.8^**^	36.1±2.9	51.3±3.7^**^
preTFT>TFT IC_75_+OHP IC_75_ 48 h	9.3±0.3^**^	50.8±4.1	39.9±4.4^*^

The cells were exposed 48 h to [IC_75_] of OHP, TFT or both drugs. These drug concentrations are mentioned in the legend of Table 2. preTFT: 4 h TFT pre-incubation period. Values are means±s.e.m. (*n*=3). Significant differences compared to OHP alone: ^*^P<0.05; ^**^P<0.01.
